# Supplementary material for: Urine metabolomic changes in cats with renal disease or calcium oxalate uroliths
Source: PLoS One. 2025 Aug 18;20(8):e0329999. doi: 10.1371/journal.pone.0329999 (PMC12360560; doi:10.1371/journal.pone.0329999)
Supplement: Attachment — Submitted filename: Response to reviewers.docx [file pone.0329999.s006.docx]

Journal Requirements:

When submitting your revision, we need you to address these additional requirements.

1. Please ensure that your manuscript meets PLOS ONE's style requirements, including those for file naming. The PLOS ONE style templates can be found at

[https://journals.plos.org/plosone/s/file?id=wjVg/PLOSOne_formatting_sample_main_body.pdf](https://track.editorialmanager.com/CL0/https:%2F%2Fjournals.plos.org%2Fplosone%2Fs%2Ffile%3Fid=wjVg%2FPLOSOne_formatting_sample_main_body.pdf/1/010f0196cc543318-c8da5917-9d02-4e8f-a582-2412fa75f1ae-000000/M_Po4eC9FLeoDRm1Lp0jgpEeBqNeIVILIEJze0RlQ38=211) and

[https://journals.plos.org/plosone/s/file?id=ba62/PLOSOne_formatting_sample_title_authors_affiliations.pdf](https://track.editorialmanager.com/CL0/https:%2F%2Fjournals.plos.org%2Fplosone%2Fs%2Ffile%3Fid=ba62%2FPLOSOne_formatting_sample_title_authors_affiliations.pdf/1/010f0196cc543318-c8da5917-9d02-4e8f-a582-2412fa75f1ae-000000/scQw_RtM2D8DesWNSKbnzNlZGurQsRM-JQ0Kf5LEdl0=211)

2. Thank you for stating the following in the Acknowledgments Section of your manuscript:

“The authors wish to acknowledge the statistical support provided by Dr. John Bredja and the excellent technical support provided by the animal care technicians of the Hill’s Pet Nutrition feline colony.”

We note that you have provided additional information within the Acknowledgements Section that is not currently declared in your Funding Statement. Please note that funding information should not appear in the Acknowledgments section or other areas of your manuscript. We will only publish funding information present in the Funding Statement section of the online submission form.

Please remove any funding-related text from the manuscript and let us know how you would like to update your Funding Statement. Currently, your Funding Statement reads as follows:

“Funding: Funded by and performed at the Pet

Nutrition Center, Hill’s Pet Nutrition, Inc., Topeka,

KS (http://www.hillspet.com/our-company.html).

The funders had no role in study design, data collection and analysis, decision to publish. The words and conclusions are uniquely the authors and not the funding agency.”

Please include your amended statements within your cover letter; we will change the online submission form on your behalf.

3. We note that the grant information you provided in the ‘Funding Information’ and ‘Financial Disclosure’ sections do not match.

When you resubmit, please ensure that you provide the correct grant numbers for the awards you received for your study in the ‘Funding Information’ section.

4. Thank you for stating the following in the Competing Interests/Financial Disclosure * (delete as necessary) section:

“We have read the journal's policy and have the following to declare: Three of the authors have an affiliation (K.S.P., S.K.T., and R.L.H) and another formerly had an affiliation (D.E.J.) to the commercial funders of this research, as employees of Hill’s Pet Nutrition, Inc.”

We note that you received funding from a commercial source: “Hill’s Pet Nutrition, Inc.”

Please provide an amended Competing Interests Statement that explicitly states this commercial funder, along with any other relevant declarations relating to employment, consultancy, patents, products in development, marketed products, etc.

Within this Competing Interests Statement, please confirm that this does not alter your adherence to all PLOS ONE policies on sharing data and materials by including the following statement: "This does not alter our adherence to PLOS ONE policies on sharing data and materials.” (as detailed online in our guide for authors http://journals.plos.org/plosone/s/competing-interests).  If there are restrictions on sharing of data and/or materials, please state these. Please note that we cannot proceed with consideration of your article until this information has been declared.

Please include your amended Competing Interests Statement within your cover letter. We will change the online submission form on your behalf.

5. Your ethics statement should only appear in the Methods section of your manuscript. If your ethics statement is written in any section besides the Methods, please delete it from any other section.

6. Please include captions for your Supporting Information files at the end of your manuscript, and update any in-text citations to match accordingly. Please see our Supporting Information guidelines for more information: [http://journals.plos.org/plosone/s/supporting-information](http://track.editorialmanager.com/CL0/http:%2F%2Fjournals.plos.org%2Fplosone%2Fs%2Fsupporting-information/1/010f0196cc543318-c8da5917-9d02-4e8f-a582-2412fa75f1ae-000000/zpRDlDqP4ie82fqg-Gp5CTsC33NZkIPYOzEwQIJZB5A=211).

Journal requirements

1 – we have reviewed the manuscript and believe it meets the PLOS ONE’s style.

2 - This funding statement should meet your needs.
**Funding:** This research was funded by Hill’s Pet Nutrition, Inc., Topeka, KS, USA (<http://www.hillspet.com/our-company.html>). Accessed April 27, 2024 which also supplied the excellent technical support of the animal care technicians.

3 – Statements have been updated as requested.

4 – Statements have been updated as requested.

5- all ethics statements are now in then methods section

6 – we have checked the captions and supporting information and it seem correct

We are including the raw data as a supplementary file to make it publicly available at publication

Additional Editor Comments:

Thank you for submitting the following manuscript to PLOS ONE.

Please revise the manuscript according to the reviewers' comments and upload the revised file.


[Note: HTML markup is below. Please do not edit.]

Reviewers' comments:

Reviewer's Responses to Questions

**Comments to the Author**

1. Is the manuscript technically sound, and do the data support the conclusions?

The manuscript must describe a technically sound piece of scientific research with data that supports the conclusions. Experiments must have been conducted rigorously, with appropriate controls, replication, and sample sizes. The conclusions must be drawn appropriately based on the data presented.

Reviewer #1: Yes

Reviewer #2: Partly

Reviewer #3: Yes

2. Has the statistical analysis been performed appropriately and rigorously?

Reviewer #1: Yes

Reviewer #2: Yes

Reviewer #3: Yes

3. Have the authors made all data underlying the findings in their manuscript fully available?

The [PLOS Data policy](http://track.editorialmanager.com/CL0/http:%2F%2Fwww.plosone.org%2Fstatic%2Fpolicies.action%23sharing/1/010f0196cc543318-c8da5917-9d02-4e8f-a582-2412fa75f1ae-000000/uDl0T3AmF3zsSncBwXEc2-BMsCEWDC-B9DY_ITqNQRk=211) requires authors to make all data underlying the findings described in their manuscript fully available without restriction, with rare exception (please refer to the Data Availability Statement in the manuscript PDF file). The data should be provided as part of the manuscript or its supporting information, or deposited to a public repository. For example, in addition to summary statistics, the data points behind means, medians and variance measures should be available. If there are restrictions on publicly sharing data—e.g. participant privacy or use of data from a third party—those must be specified.

Reviewer #1: No

Reviewer #2: Yes

Reviewer #3: No

4. Is the manuscript presented in an intelligible fashion and written in standard English?

PLOS ONE does not copyedit accepted manuscripts, so the language in submitted articles must be clear, correct, and unambiguous. Any typographical or grammatical errors should be corrected at revision, so please note any specific errors here.

Reviewer #1: Yes

Reviewer #2: Yes

Reviewer #3: Yes

5. Review Comments to the Author

Please use the space provided to explain your answers to the questions above. You may also include additional comments for the author, including concerns about dual publication, research ethics, or publication ethics. (Please upload your review as an attachment if it exceeds 20,000 characters)

Reviewer #1: This is a very interesting and good organized research. Renal disease (RD) and
calcium oxalate (CaOx) stones are most important problems on cat . The objective of this study was to assess urine
metabolomic parameters of cats with these diseases to determine metabolic
abnormalities and differences between the groups. For these reasons;
1. Please give more details about urine and hematological/biochemical results for healthy and sick cats. How did you do the diagnose? Inorganic phosphorus, BUN, Creatinine, urine microscobic analyses, Protein levels, glucose and the position of anemia, WBC and PLT? What about correlations between these parameters and metabolites?

The hematological/biochemical results were previously reported in: Jewell DE, Tavener SK, Hollar RL, Panickar KS. Metabolomic changes in cats with renal disease and calcium oxalate uroliths. Metabolomics. 2022 Aug 13;18(8):68. doi: 10.1007/s11306-022-01925-4. PMID: 35962261; PMCID: PMC9374649. We have added the details associated with assignment to treatments in the methods section. We thought the paper was sufficiently lengthy without the correlations, although we are making the data publicly available so that could be followed up should it be desired.
2.Did you do any microbiological analysis on urine and ?f you have them please give the results do any microbiological analysis.

We did not evaluate any microbiological analysis.
3. Did you know anything about viral infections?

We did not evaluate any viral components.
4. Do you have any news about the etiological factors of Renal disease and Calcium oxalate

We have added a paragraph in the discussion regarding the etiology of these diseases.
5. Inclusion and exclusion criterias of your cases? I can not see Table S1 and S2.

We have added the criteria for inclusion and exclusion and reformatted S1 and S2.
6. What about nutrition of your cases?

Unfortunately, the nutrition was quite variable as a function of the retrospective aspect of this study. Most of the time the cats were eating a variety of foods which changed often as they were used in palatability studies.
7. Your introduction is so short. You should more different literatures such as:
The clinical efficacy of cGMP-specific sildenafil on mitochondrial biogenesis induction and renal damage in cats with acute on chronic kidney disease. M Maden, M Ider, ME Or, B Dokuzeylül, E Gülersoy, MC K?l?çkaya, B Bilgiç, ...(2024)
BMC Veterinary Research 20 (1), 499
Clinical efficacy of marbofloxacin in dogs and cats diagnosed with lower urinary tract disorders. B Dokuzeylül, B Celik, BD Si?irci, BB Kahraman, SÜ Saka, A Kayar, S Ak, ..(2019) Med. Weter 75 (9), 549-552

The introduction was enhanced with a broader discussion as you suggested. We included the first article but the second seemed out of scope as it didn’t specifically deal with either condition. It is interesting to speculate that infections are a risk factor for both conditions, but the literature support seems weak.

Reviewer #2: PONE-D-25-17831 Review comments
Urine metabolomic changes in cats with renal disease or calcium oxalate uroliths
This well-structured study provides valuable insights into urine metabolomic profiles in cats with renal disease (RD) and calcium oxalate (CaOx) uroliths. The use of repeated urine samples, creatinine normalization, and robust data reduction through factor analysis strengthens the dataset and supports the identification of disease-specific metabolic patterns. The findings are intriguing and suggest that urinary metabolites offer more than a passive reflection of serum changes, potentially revealing functional kidney alterations.
Strengths:
Clear research objectives with appropriate rationale.
Inclusion of both RD and CaOx groups with healthy controls for comparative profiling.
Longitudinal sampling per cat and correct statistical handling of repeated measures.
Application of factor analysis to reduce data complexity and focus on key metabolic features.
Proper normalization of analyte concentrations to urinary creatinine.
Identification of novel metabolic signatures, particularly involving purine nucleotides and phospholipids.
Areas for Improvement:
Disease Characterization: The lack of specific details regarding the type, stage, and severity of RD, as well as the composition of CaOx stones, limits the interpretation and comparability of the findings.

We have added the specific details regarding the RD group and highlighted the previous report of these in the publication dealing with changes in circulating concentrations. All stones were analyzed as CaOx which has been added as well.
Factor Interpretation and Biological Significance: The biological interpretation of the identified factors and the underlying metabolic pathways needs significant expansion. This includes explaining the elevation of certain metabolites in specific groups and integrating the findings with known feline physiology, diet, and the pathophysiology of RD and urolithiasis in cats and other species.

Discussion was enhanced as requested.
Graphical Presentation: The inclusion of figures (e.g., heatmaps,	o plots, pathway enrichment maps) is needed to enhance clarity and engagement.

We looked at heat maps, volcano plots, and pathway enrichment maps, and looking at the data in the manuscript it did not seem that volcano plots or pathway enrichment maps are relevant to this manuscript. Perhaps another study working on gene expression, not metabolite differences between disease groups would fit better. Table 2 in the manuscript is a short table, and probably has no advantage if shown as a heat map. Table 3 presents the factor loadings and thus a heat map would not be relevant to this table either. In addition, some color coding is already used with Table 3 in that negative loadings are presented in red font, and positive loadings in black font. This leaves the supplemental tables. Supplemental Table S2 presents the eigenvalues and percent variance explained. These are not the kind of results with heat maps. Supplemental Table S3 is again the factor loadings, just like manuscript Table 3, but for all factors, not just statistically significant factors. Finally then there is supplemental table S1. There is a lot of information in this table, and itd seemed that the color coding of a heat would aid interpretation. So, we have enhanced the supplementary file to make it in the heatmap form.

Causality vs. Association: While the study shows associations, it's crucial to acknowledge that it doesn't establish causality between metabolic alterations and disease pathogenesis.
Terminology and Language: Ensure consistent use of terms (e.g., RD vs. CKD) and address minor grammatical issues.

We have added a discussion of causality as compared to association – that is a significant understanding and is good to include. Checked for consistent use of terms and grammar.

Reviewer #3: I would like to congratulate the authors on the interesting and relevant study, which raises important hypotheses regarding urinary metabolomic alterations associated with renal disease and calcium oxalate urolithiasis in felines. Below, I present some comments and suggestions.

The attached table was provided in Word format, making it impossible to view all essential clinical data such as age, urinary creatinine, and other parameters.

We are sorry this was not available. We have changed the format which should make it able to be viewed.

Line 23: The description of the factors is overly detailed and may hinder understanding for clinical readers. It is suggested to summarize the main findings with a focus on the most relevant clinical/metabolic implications.

We have added discussion to emphasize what seemed like the most important changes of the factors.

Line 45: The small sample size of the reference limits the epidemiological relevance of the study. It is recommended to use additional or more robust sources for context.

Unfortunately, we have exhausted our available feline RD and CaOx stone formers available to us for this study so no more could be added. We have noted this limitation and added that note to the manuscript.

Line 50: Although not the focus of the study, struvite and diet are mentioned without addressing dietary mechanisms related to oxalate stones, which are central to the manuscript.

We have added a discussion regarding the dietary mechanisms of calcium oxalate stone formation.

Lines 61–63: It would be enriching to briefly contextualize the role of	, especially in the areas of feline nephrology and urolithiasis. It would be interesting to highlight current knowledge gaps and justify the choice of the metabolomic approach as a diagnostic and research tool.

An increased discussion of the use of the metabolomic approach was added as requested.

Line 68: Information on environmental management and inclusion criteria for experimental groups is vague. It is recommended to include details such as type of diet, age range, and diagnostic criteria used for group classification (e.g., imaging, clinical history, laboratory data).

This was added as well and we highlighted the previously published reference that discussed these changes dealing with the circulating concentration in these pets.

Line 80: There is no detail regarding the method of urine collection, measures to avoid contamination, or average storage time before analysis.
The methodological description relies heavily on external references. It is recommended to specify the number of technical replicates, the use of quality controls, and the criteria for data exclusion.

Enhanced methodological description was added.

The “annual and end-of-life” urine collection lacks a more precise definition regarding the timing within the renal disease course. It would be important to detail the intervals between collections and the clinical stage of the animals at the time of sampling.

This is difficult as the precise timing of renal disease and loss of renal function is not known. Pets were euthanized when their quality of life was so degraded the veterinary staff concluded it was best for the pet. There is no known interval because of this and that has been added as a limitation.

Line 310: The limitation of the sample to a cat colony restricts the generalization of the findings, considering the low genetic, environmental, and nutritional variability. This aspect could be further emphasized in the study’s limitations.

This has now been further discussed in the limitations.
The manuscript suggests that the increase in urinary inosine in cats with CaOx stones may reflect an inflammatory process. However, inflammatory biomarkers (such as cytokines) that would support this hypothesis were not evaluated. Although the cited literature suggests an immunomodulatory role of inosine, extrapolation to felines remains speculative. A more cautious reformulation of the interpretation is recommended, highlighting the absence of direct evidence.

We have added this to the further discussed limitations.

The proposal that increased urinary BHBA may be beneficial due to its anti-inflammatory effects is interesting, but based on studies in other species (e.g., rats). It is suggested to present this interpretation as a hypothesis to be tested and not as a direct inference, reinforcing the need for controlled clinical studies in cats.

We have added this to the further discussed limitations.

The practice of normalizing urinary concentrations by creatinine assumes stable excretion of this substance. This premise may be questionable in cats with renal dysfunction. It is recommended to present absolute urinary creatinine data between groups to verify the validity of this correction.

Unfortunately, because of the retrospective foundation of this study, all we had available were the banked urine samples. We are unable to measure the actual total creatinine excretion per day of these cats. It seems clear that the changes associated with the analyte to creatinine ratio is a better estimate of actual clearance than using the concentration in urine when we had previously shown that the expected urine dilution (and thus lower urine specific gravity) was present in the RD and to a lesser extent CaOx cats this is the reference 27 Jewell et al, 2022.

6. PLOS authors have the option to publish the peer review history of their article ([what does this mean?](https://track.editorialmanager.com/CL0/https:%2F%2Fjournals.plos.org%2Fplosone%2Fs%2Feditorial-and-peer-review-process%23loc-peer-review-history/1/010f0196cc543318-c8da5917-9d02-4e8f-a582-2412fa75f1ae-000000/imPDcEg6JT2GR4JQvG-a43awNLgoX2zm7qv1vvHQep4=211)). If published, this will include your full peer review and any attached files.


If you choose “no”, your identity will remain anonymous but your review may still be made public.


**Do you want your identity to be public for this peer review?** For information about this choice, including consent withdrawal, please see our [Privacy Policy](https://track.editorialmanager.com/CL0/https:%2F%2Fwww.plos.org%2Fprivacy-policy/1/010f0196cc543318-c8da5917-9d02-4e8f-a582-2412fa75f1ae-000000/TCzwaukuJOxYFr4z_9Eu9jn62ABV53n5sSmWSUlk-Ug=211).

Reviewer #1: No

Reviewer #2: No

Reviewer #3: **Yes:** MARIA EDUARDA GONÇALVES TOZATO

[NOTE: If reviewer comments were submitted as an attachment file, they will be attached to this email and accessible via the submission site. Please log into your account, locate the manuscript record, and check for the action link "View Attachments". If this link does not appear, there are no attachment files.]

While revising your submission, please upload your figure files to the Preflight Analysis and Conversion Engine (PACE) digital diagnostic tool, [https://pacev2.apexcovantage.com/](https://track.editorialmanager.com/CL0/https:%2F%2Fpacev2.apexcovantage.com%2F/1/010f0196cc543318-c8da5917-9d02-4e8f-a582-2412fa75f1ae-000000/TTEDPmu31-Mib0nzhrwFU2j8YU5OtqJ1OuCam-VRRos=211). PACE helps ensure that figures meet PLOS requirements. To use PACE, you must first register as a user. Registration is free. Then, login and navigate to the UPLOAD tab, where you will find detailed instructions on how to use the tool. If you encounter any issues or have any questions when using PACE, please email PLOS at [figures@plos.org](mailto:figures@plos.org). Please note that Supporting Information files do not need this step.
